# Fertility Outcomes and Sperm-DNA Parameters in Metastatic Melanoma Survivors Receiving Vemurafenib or Dabrafenib Therapy: Case Report

**DOI:** 10.3389/fonc.2020.00232

**Published:** 2020-03-05

**Authors:** Marco Ghezzi, Andrea Garolla, Sabina Magagna, Iva Šabovich, Massimiliano Berretta, Carlo Foresta, Luca De Toni

**Affiliations:** ^1^Department of Clinical and Experimental Oncology, IOV-IRCCS, Padua, Italy; ^2^Unit of Andrology and Reproductive Medicine, Department of Medicine, University of Padua, Padua, Italy; ^3^Department of Medical Oncology, National Cancer Institute, IRCCS Aviano, Aviano, Italy

**Keywords:** metastatic melanoma, BRAF inhibitors, male fertility, sperm DNA, cryopreservation

## Abstract

Melanoma is a frequent neoplasm in young adult males in reproductive age, 10% of them degenerating into regional and/or distant metastases (MM). The use of BRAF inhibitors (BRAFi) vemurafenib and dabrafenib is effective in MM patients harboring *BRAF* V600E/K/D mutations. Despite the increased life expectancy in MM patients treated with BRAFi, concerns are raised by the possible side effects and increased risk of gonado- and/or genotoxicity associated with these drugs. However, these aspects are currently under-investigated. Here we report the different fertility outcome in two cases of MM patients, harboring *BRAF* V600E mutation, that received vemurafenib and dabrafenib respectively. The first patient, 36 years at recruitment in 2015 and seeking fatherhood, had an history of relapsing melanoma since 2002 and undergone to numerous interventions and chemotherapy cycles. In November 2011, following detection of *BRAF* V600E mutation, a daily treatment with vemurafenib (1,440 mg) was prescribed with preventive gamete cryopreservation. BRAFi was effective in the clinical stabilization of the disease. In 2015, semen evaluation at follow-up showed sperm parameters within the normal range and no signs of alteration of either sperm function or sperm-DNA. On these bases, no contraindications for fatherhood were given. After a month of free intercourses, the 38-year-old partner achieved spontaneous pregnancy with a regular course, normal male fetal karyotype and a full term birth. The second patient, 39 years at recruitment in 2018 and seeking fatherhood, had an history of melanoma since 2012. In 2018, following the evidence of disease relapse and detection of the *BRAF* V600E mutation, treatment with dabrafenib/trametinib (300 mg/day/2 mg/day) was initiated together with preventive gamete cryopreservation. In 2019, semen evaluation at follow up showed sperm count and motility below the reference values, associated with increased indexes of sperm aneuploidies and sperm DNA fragmentation. Accordingly, access to assisted reproduction technique with cryopreserved spermatozoa was suggested. Differently from dabrafenib that was associated to damage to spermatogenesis, high-dose vemurafenib showed no association with gonadotoxicity and genotoxicity in humans, even at high doses. Although further confirmation are required, our data represent a valued cue in oncofertility counseling to MM patients in addition to preventive cryopreservation.

## Background

The increasing availability of therapeutic options represent a challenging issue in comprehensive cancer care. In fact, in face of an undoubted advantage deriving from having a greater arsenal of molecules, long term quality-of-life of cancer survivors may result affected by these novel drugs or clinical approaches, raising concerns of paramount importance. This is particularly the case for melanoma, representing the most common cancer diagnosed in patients in the age range of 25–29 years (1). To this regard, a negative prognostic factor in melanomas is represented by mutations of *BRAF* gene, coding for a serine-threonine protein kinase involved in mitogen-activated protein kinase (MAPK) pathway. Cancer cells harboring V600E or V600K *BRAF* mutations result in a constitutively active signaling pathway, driving to cell proliferation and metastasis (2, 3). Activating mutations of *BRAF* gene are detected in almost half of melanomas, 80–90% of which are represented by V600E mutation whilst 10–20% are V600K mutation (4–6). In this framework, the class of drugs belonging to BRAF inhibitors (BRAFi) bind to the ATP-binding site of mutated BRAF protein promoting the stabilization it into an active conformation. As a consequence, because of the missed free access to ATP to its binding site, the downstream activation of MAPK pathway results turned off (7). Vemurafenib and dabrafenib are two of the available BRAFi and represent the first-line adjuvant chemotherapy in metastatic melanoma harboring BRAF mutation. In particular, the use of dabrafenib is associated with 50–59% response rate and 5.5–6.3 months progression-free survival, whereas vemurafenib showed the 63% reduction in the risk of death and a 74% reduction in the risk of either death or disease progression (8).

The use of BRAFi is associated to class-specific adverse effects (AEs), such as rash, hair loss, photosensitivity reactions, keratinocytic proliferation, gastro-intestinal disorders, and hematological events (9). In addition, significant impact on reproductive potential in both males and females has been described as BRAFi-related AEs. Available studies in animal models revealed potentially irreversible spermatogenesis damage after low doses dabrafenib administration, as well as reduced corpora lutea in pregnant rodents. In addition, dabrafenib showed to be teratogen in pre-clinical studies by severely affecting embryonic/fetal development (10). So far, there is less experience about the fertility issues associated with the use of vemurafenib, considering also the substantial lack of studies in humans (11). On these bases, gametes cryopreservation is prudently suggested during early oncological counseling to melanoma patients, even at the time of early diagnosis, in order to allow fertility seeking on later stage of the disease or at remission (11).

Here we provide the case report of two male patients with a primary diagnosis of local melanoma, subsequently degenerated into invasive-metastatic phenotype associated with BRAF mutation. Adjuvant therapy with vemurafenib and dabrafenib, respectively in the first case and in the second case presented, was then administrated, resulting in disease-free survival. The fertility potential of patients and the eventual achievement of natural or assisted child birth were then recorded.

## Case Presentation

### Patient 1

A young man aged 36 came to our observation at the Unit of Andrology and Reproductive Medicine (Padova, Italy) in 2015 seeking fertility. The patient did not refer any major abnormality at birth (no under- or overweight, no undescended testis, no preterm birth, no metabolic derangements). In addition, in adulthood no varicocele or other urogenital pathologies were reported.

Previously in 2002, the patient undergone to the excision of an ulcerated left paravertebral lesion, successively identified as melanoma with presence of numerous mitosis and negative biopsy of sentinel lymph node.

From 2002 to 2007 he reported healthy conditions. In 2007, axillary lymph node was investigated for diagnosis purposes and then removed. The subsequent analysis confirmed the detection of melanoma metastasis. From this diagnosis onwards, the patient undergone to frequent surgical and chemotherapy interventions due to subsequent relapses such as: ileal resection with lymphadenectomy in 2009; gamma-knife on frontal and parietal metastasis intercalated to medical therapies (fotemustine) in 2009; exeresis and electrochemotherapy of thoracic skin lesions in 2010; treatment with ipilimumab between July and September 2010; treatment with interferon and temozolomide in 2010 and 2011; stereotactic radiosurgery on metastasis of the left temporal-occipital cortical sulcus, associated with tonsillectomy and removal of a laterocervical lesion, in 2011. The patient reported no semen evaluation before the beginning of any cytotoxic chemotherapy.

On November 2011, following the confirmation of disease progression for 4 years, the *BRAF* V600E mutation was detected and the treatment with vemurafenib was initiated at a dosage of 1,440 mg/day. The patient referred no remarkable adverse events due to drug dosing, excepted of accentuated photosensitivity, resulting in overall improvement of the disease and stabilization of clinical status. In agreement with the initiation of the treatment with vemurafenib, preventive cryopreservation of spermatozoa was performed. Semen parameters at both the time of cryopreservation and at follow-up in 2015, after 4 years of treatment with vemurafenib, are reported in Table 1. The patient showed semen parameters, such as sperm count, sperm motility, and cell viability, above the reference values for normal fertility at both basal and follow-up. In order to address any possible influence of BRAFi on sperm DNA status, the evaluation of sperm aneuploidies and fragmentation of sperm DNA were also assessed, finding no additional impairment of these specific parameters. The hormonal pattern of testis function, such as serum total testosterone and luteinizing hormone levels, were within the normal-ranges. However, vitamin D insufficiency with no secondary elevation of parathormone levels and a subclinical hypothyroidism were detected, requiring a subsequent follow-up every 6 month. On the base of the observed normal semen parameters at follow-up, no contraindications were given on regard to seek for natural fertility on November 2015. Since then, after about 1 month of attempts, the partner (38 years old) reported spontaneous pregnancy with a regular course and normal amniocentesis for male fetal karyotype. The pregnancy ended with preterm cesarean section at the gestational age of 8 months for membrane rupture. At birth, the baby weighed 2,100 g showed absence of major abnormalities.

**Table 1:** Semen, sperm DNA and hormonal parameters at recruitment and at follow up of the two patients undergone to adjuvant therapy Vemurafenib and Dabrafenib, respectively.

	**Patient 1 (Vemurafenib)**		**Patient 2 (Dabrafenib)**	
	**Values at cryopreservation**	**Values at follow-up**	**Values at cryopreservation**	**Values at follow-up**
**SEMEN PARAMETERS**				
**Sperm concentration** (Reference value: > 15 ×10^6^cells/mL)	84	90.3	45	25
**Total sperm count** (Reference value: > 39 ×10^6^ cells)	126	225.8	54	**12.5**
**Progressive motility** (Reference value > 32%)	66	58	**28**	**18**
**Sperm viability** (Reference value: > 58%)	89	90	76	76
**Sperm with normal morphology** (Reference value: > 4%)	20	18	10	6
**SPERM DNA PARAMETERS**				
**Total sperm aneuploidies** (Reference value: <1.5%)	0.89	0.91	1.21	1.48
**Sperm DNA condensation - Aniline test** (Reference value: ≥45%)	//	67	//	62
**Early sperm apoptosis – Annexin V test** (Reference value: % N/A)	//	81	//	75
**Sperm mitochondrial function - JC-1 test** (Reference value: ≥75%)	//	91	//	**65**
**Sperm chromatin heterogeneity - Acridine orange test** (Reference value: ≥70%)	//	82	//	71
**Sperm DNA fragmentation - TUNEL test** (Reference value: ≥80%)	//	93	//	**72**
**Sperm DNA double-strand breaks – γH2AX test** (Reference value: % N/A)	//	95	//	84
**HORMONAL PARAMETERS**				
**Total testosterone** (Reference value: 10.4–29.0 nmol/L)	//	21.41	//	**30.33**
**LH** (Reference value: 1–8 IU/L)	//	6.9	//	6.1
**FSH** (Reference value: 1–8 IU/L)	//	7.3	//	7.1
**FT4** (Reference value: 9–22 pmol/L)	//	15.67	//	13.47
**TSH** (Reference value: 0.4–4.0 μU/L)	//	**4.32**	//	0.85
**25 OH vitamin D** (Reference value: > 50 nmol/L)	//	**16**	//	**34**
**PTH** (Reference value:4.6–26.8 ng/L)	//	19.9	//	10.2

*LH, luteinizing hormone; FSH, follicle stimulating hormone; FT4, free thyroxine; TSH, thyroid stimulating hormone; PTH, parathormone; N/A, not available. Values outside normal ranges are in bold. //, not available*.

### Patient 2

A man aged 39 came to our observation at the Unit of Andrology and Reproductive Medicine in 2018 seeking fertility. The patient did not refer any major abnormality at birth (no under- or overweight, no undescended testis, no preterm birth, no metabolic derangements). In addition, in adulthood no varicocele or other urogenital pathologies were reported. In 2012 the patient undergone the excision of a right lower limb melanoma, with inguinal-iliac loco-regional lymph nodes dissection for metastasis localization. Thereafter, the patient displayed a disease-free course and, in 2014, he decided to seek fertility. After few months of free sexual intercourse, the partner (37 years old) reported spontaneous pregnancy with regular course and delivery of a male newborn with no apparent major abnormalities.

In 2016, the patient showed a disease loco-regional relapse and undergone to total right lower limb perfusion with melphalan and tumor-necrosis factor with no other health problem and disease-free survival until 2018. In March 2018, following the evidence of disease progression and detection of the *BRAF* V600E mutation, he was scheduled for dabrafenib/trametinib therapy. Before starting BRAFi treatment, a preventive cryopreservation of spermatozoa was performed together with semen analysis and evaluation of sperm aneuploidies status. Basal semen parameters, at the time of cryopreservation, are summarized in Table 1. Sperm count and viability showed values within the normal range of fertility with the exception of moderate low levels of cell motility accounting for asthenozoospermia. On April 2018, the treatment with dabrafenib at the dosage of 300 mg/day, associated with MEK-inhibitor trametinib (2 mg/day), was initiated. The patient referred no adverse events due to drug dosing, resulting in overall improvement of the disease and stabilization of clinical status.

On March 2019, during the course of dabrafenib/trametinib therapy, a control of the fertility status was performed by semen analysis whose results are reported in Table 1. A significant reduction of total sperm count to levels below the reference values was observed, together with both the reduction of sperm motility index and the percentage of sperm cells bearing chromosomal aneuploidies. In addition, the analysis of sperm DNA fragmentation indexes showed higher values of single strand breaks, outlined by TUNEL test, with respect to reference. In regard of the testis endocrine function, total serum testosterone and luteinizing hormone were within in the normal-range. However, low serum levels of vitamin D, compatible with insufficiency status were detected. On this base, and in relation to the desire of another pregnancy, the couple was suggested to access assisted reproduction technique with use of cryopreserved spermatozoa.

## Investigations

The study was performed in accordance with the Declaration of Helsinki protocols. All patients provided their written informed consent prior to the preparation of this case report.

Semen samples were obtained by masturbation after 2–5 days of sexual abstinence. After liquefaction at room temperature, semen volume, pH, sperm concentration, total sperm count, viability, motility, and normal morphology were determined according to World Health Organization guidelines for semen analysis (12). Semen samples were then washed three times with sterile phosphate-buffered saline, and the pellet was used for the subsequent analyses.

The study of sperm aneuploidy was performed by multicolour FISH, as reported elsewhere (13). The DNA hybridization was performed using a human satellite probe-specific mix for autosomes 13, 18, 21 and sex chromosomes X, Y (Kreatech Diagnostics, Amsterdam, The Netherlands). Probes were directly labeled with a specific fluorochrome for each chromosome. The protocol of FISH staining, including sperm nucleus decondensation, DNA denaturation of sperm, incubation with probes, post-hybridization washing and nuclear counter-staining with 6-diamino-2-phenylindole (DAPI), was performed according to the manufacturer's indications. Slides were finally evaluated with the use of a fluorescence microscope (Nikon Eclipse E600) equipped with a triple band-pass filter set. Single spots were evaluated as reported elsewhere (13). For each patient, at least 2,500 cells were scored.

Evaluation of sperm DNA condensation index through aniline test, early sperm apoptosis index through Annexin V test, sperm mitochondrial function index through JC-1 test, sperm chromatin heterogeneity index through acridine orange test, sperm DNA fragmentation index through TUNEL test and sperm DNA double-strand breaks index through γH2AX test were performed as described elsewhere (14).

Blood samples were collected in the fasting state between 08.00 and 10:00 h. Serum follicle-stimulating hormone, luteinizing hormone and testosterone were evaluated by commercial electrochemiluminescence immunoassay methods (Elecsys 2010; Roche Diagnostics, Mannheim, Germany) as reported elsewhere (15, 16). Serum parathormone levels were determined with a direct, two-site, sandwich type chemiluminescent immunoassay (LIAISON N-TACT PTH, DiaSorin Inc. Stillwater, MN). Serum 25(OH) Vitamin D was determined with direct, competitive chemiluminescent immunoassay (LIAISON 25 OH Vitamin D TOTAL Assay, DiaSorin Inc). Serum thyroid-stimulating hormone was measured using a chemiluminescent immunoassay on the Modular Analytics E170 (Roche Diagnostics GmbH, Mannheim, Germany).

## Discussion

With the aim to manage the possible fertility-threatening issues associated to medical therapy and/or surgery treatment for cancer disease, oncofertility is a new interdisciplinary field gaining more and more attention particularly in those branches of oncology recording major successes in terms of disease-free survivals (17–19). As an example, we recently evaluated the effect of chemo- and radiotherapy as adjuvant treatments for testicular cancer, another oncological disease affecting males at reproductive age. We observed that both treatments were equally associated with transient impairment of semen parameters, but long-term increase of sperm aneuploidies and impaired endocrine function of the testis, leading to subclinical hypogonadism and vitamin D deficiency that appeared as matter of concern (16, 20). In fact, in addition to infertility, these conditions represent independent risk factors for cardiovascular disease and osteoporosis respectively. Therefore, an adequate oncological counseling to cancer patients appears as mandatory in order to correctly address the risk/benefit ratio of available adjuvant therapies (20–27).

In the present case reports we provide evidence that adjuvant therapy with BRAFi dabrafenib and vemurafenib in male metastatic melanoma survivors associates with a differential impact of semen quality according to the specific drug molecule of choice. In particular, dabrafenib was associated with higher gonadal toxicity compared to vemurafenib, in terms of reduction of sperm count, motility, and possibly DNA fragmentation. Importantly, the two patients were highly comparable in terms of age and clinical history: from original diagnosis and treatment of the primary tumor and subsequent relapses, to detection of *BRAF* mutation and follow-up of the therapy with BRAFi, including evaluation of semen parameters. These elements allow to rule out a major involvement of the patients background on reproductive issues.

Our data gain particular value in the light of the paucity of studies on the reproductive toxicity of this class of drugs. Indeed, despite sharing the same molecular target, vemurafenib and dabrafenib display several differences at both pharmacodynamics and pharmacokinetic level. In fact, in most of the cell lines with *BRAF* mutations, IC_50_ of vemurafenib is nearly one order of magnitude greater than that observed for of dabrafenib (<1,000 nmol/L vs. <100 nmol/L, respectively) (28). In addition, according to the biopharmaceutics classification system, vemurafenib is classified with low solubility-low permeability properties, differing from the low solubility-high permeability classification of dabrafenib (29–31). On these bases, it is reasonable to expect that the anti-proliferative effects of these two drugs may reverberate differentially on the reproductive function and in particular on spermatogenesis which is highly sensitive to environmental noxae (32). To this regard, dabrafenib has been classified in the D rating for the pregnancy-risk category (33) with evidence of fertility issues. In fact dabrafenib showed teratogenic and embryotoxic effects in rats when used at threefold dosages compared to those used in humans at the recommended clinical dose. In addition studies on male rats and dogs, using a fifth of the dosage associated with the human clinical exposure, showed significant testicular impairment that persisted in a 4-week recovery period (10, 11). On the other hand, vemurafenib received B rating without evidence of gonadal toxicity and no available data in humans. However, preclinical studies in male and female mammalian models were performed at dosages corresponding to nearly the 10% of the anticipated clinical exposure in humans (10, 11). No consideration can be made for trametinib, for which there is almost complete absence of data on fertility issues, that however received B rating (11). Available data in humans mainly rely on a recent case report from Cocorocchio et al., showing natural fertility in a 29 years old male patient, with *BRAF* V600E mutated metastatic melanoma, who fathered after a 2 years treatment with Dabrafenib and Trametinib (34). Importantly, authors reported limited alterations in sperm parameters evaluated 6 months before conception (1.5 years of treatment with Dabrafenib and Trametinib). However, major methodological differences can be outlined by the comparison with our study, particularly in regard of the use of reference values for semen analysis, more stringent in our case, and a more complete evaluation of sperm status that included the investigation of DNA fragmentation and chromosomal disorders.

Acknowledging the limit of case reports, data presented here represent an important oncofertility issue for male metastatic melanoma survivors, that should be carefully considered when starting an adjuvant therapy with BRAFi. Given for granted that preventive sperm cryopreservation represents the first-choice approach to preserve fertility in those patients starting the clinical course for the treatment of melanoma, it is our opinion that these data should be taken into consideration during oncological counseling in melanoma patients. This is particularly the case of younger subjects, predicted to display a better response to adjuvant chemotherapy and a favorable survival prognosis and, for these reasons, expected to resume a long-term fertility seeking.

In conclusion, despite more studies performed in different centers with larger sample sizes are needed to confirm our data and to possibly identify different side effects of the two drugs on other organs, this report represents a valued cue in oncofertility counseling to patients affected by dermatological malignancies.

## Ethics Statement

Collection of biological samples were conducted following written informed consent from studied individuals. The study was performed in accordance with the Declaration of Helsinki protocols.

## Author Contributions

SM and IŠ performed semen analysis and the assessment of sperm aneuploidies, sperm function, and sperm-DNA integrity. MB and CF supervised manuscript drafting. AG critically discussed the case history presentation. MG clinically managed the patients. MG and LD designed the study an drafted most of the manuscript.

### Conflict of Interest

The authors declare that the research was conducted in the absence of any commercial or financial relationships that could be construed as a potential conflict of interest.
